# Structural and mechanical properties of folded protein hydrogels with embedded microbubbles†

**DOI:** 10.1039/d2bm01918c

**Published:** 2023-02-08

**Authors:** Christa P. Brown, Matt D. G. Hughes, Najet Mahmoudi, David J. Brockwell, P. Louise Coletta, Sally Peyman, Stephen D. Evans, Lorna Dougan

**Affiliations:** a School of Physics and Astronomy, Faculty of Engineering and Physical Sciences, University of Leeds Leeds UK L.Dougan@leeds.ac.uk; b ISIS Neutron and Muon Spallation Source, STFC Rutherford Appleton Laboratory Oxfordshire UK; c Astbury Centre for Structural Molecular Biology, University of Leeds Leeds UK; d School of Molecular and Cellular Biology, Faculty of Biological Sciences, University of Leeds UK; e Leeds Institute of Medical Research, Wellcome Trust Brenner Building, St James's University Hospital Leeds UK

## Abstract

Globular folded proteins are powerful building blocks to create biomaterials with mechanical robustness and inherent biological functionality. Here we explore their potential as advanced drug delivery scaffolds, by embedding microbubbles (MBs) within a photo-activated, chemically cross-linked bovine serum albumin (BSA) protein network. Using a combination of circular dichroism (CD), rheology, small angle neutron scattering (SANS) and microscopy we determine the nanoscale and mesoscale structure and mechanics of this novel multi-composite system. Optical and confocal microscopy confirms the presence of MBs within the protein hydrogel, their reduced diffusion and their effective rupture using ultrasound, a requirement for burst drug release. CD confirms that the inclusion of MBs does not impact the proportion of folded proteins within the cross-linked protein network. Rheological characterisation demonstrates that the mechanics of the BSA hydrogels is reduced in the presence of MBs. Furthermore, SANS reveals that embedding MBs in the protein hydrogel network results in a smaller number of clusters that are larger in size (∼16.6% reduction in number of clusters, 17.4% increase in cluster size). Taken together, we show that MBs can be successfully embedded within a folded protein network and ruptured upon application of ultrasound. The fundamental insight into the impact of embedded MBs in protein scaffolds at the nanoscale and mesoscale is important in the development of future platforms for targeted and controlled drug delivery applications.

## Abstract 2

Globular folded proteins are powerful building blocks to create biomaterials with mechanical robustness and inherent biological functionality.

## Graphical abstract

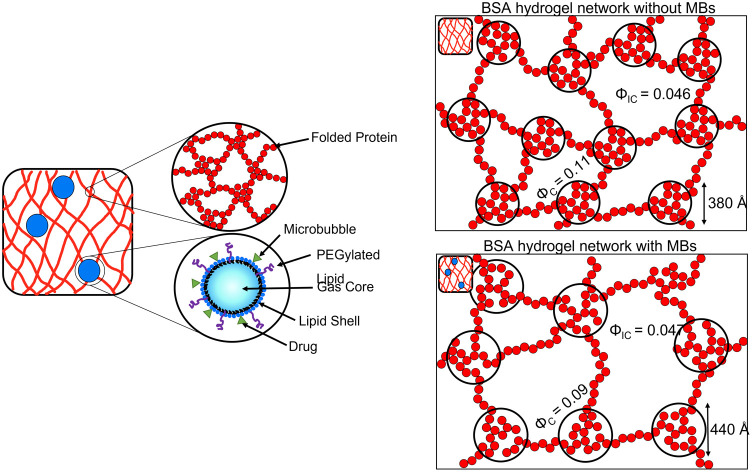

## Introduction

**Fig. 1:**
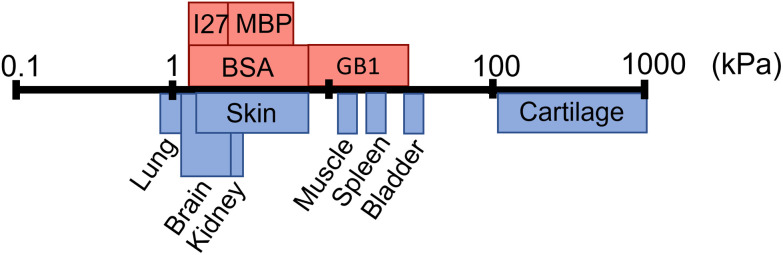
Shear modulus (*G*′) of different biological tissues (blue) and the *G*′ of folded protein hydrogels (red) including bovine serum albumin (BSA), maltose binding protein (MBP), guanine nucleotide-binding protein (GB1), and interleukin-27 (I27), where the data is collated from multiple sources.^4–17^

Folded proteins are vital in biological processes, where they provide diverse functionality and mechanical properties, which span numerous length scales.^1–3^ In addition to their naturally encoded function and structure, proteins are inherently biocompatible, making them a desirable component for biomaterial applications.^18,19^ Proteins are used as the building blocks of cross-linked hydrogel networks, where protein hydrogels have been used for a wide range of applications including tissue engineering,^20,21^ drug delivery^22,23^ and cell mechanobiology.^24^ Though many studies have investigated protein hydrogels, few have incorporated the functionality of the protein fold to create a smart biomaterial. The so-called folded protein hydrogels form hydrogel networks *via* chemical crosslinking of globular folded proteins, providing the potential to utilise the unique functions of proteins.^17,25–29^ Previous studies have shown that folded protein hydrogels are responsive to external stimuli such as chemical modulation and light exposure,^30^ have programmable shape changes,^31^ are responsive to the hydrogel solute environment,^32^ as well as the ability to control and preserve mechanical properties.^33–36^ Changes to the protein, or the cross-linking procedure, can lead to modulation of the resultant network mechanical properties, allowing for tuneable stiffness in protein hydrogels.^13,37–39^ Recently, the stiffness of bovine serum albumin (BSA) hydrogels was controlled *via* inducing protein unfolding.^14^ Dithiothreitol (DTT) breaks the nano-staples within the folded protein. By preparing BSA hydrogels with DTT, unfolding of the BSA monomers is induced, resulting in an increase in the stiffness of the bulk BSA hydrogel. The resultant structure of folded protein hydrogels from small angle X-ray and neutron scattering (SAXS and SANS) showed a heterogeneous mesoscale structure with fractal-like clusters of folded proteins connected by inter-cluster regions of unfolded proteins. By manipulating the force lability of the protein building block, the protein can be unfolded, leading to mesoscale changes to the protein network structure, including the dimensions of the cluster and inter-cluster regions, creating space in which drugs could be embedded.^6^ Furthermore, for photo-initiated BSA hydrogels, the hydrogel stiffness can be increased by increasing the rate of chemical cross-linking, controlling the diffusion- and reaction limited aggregation of the protein.^37^ These examples demonstrate that globular, folded proteins provide a functional building block with the potential for tuneable hydrogels to suit a range of applications. Fig. 1 demonstrates that even single folded protein hydrogels can be tuned to have stiffness in the range of 1–50 kPa, with the potential of tailoring the mechanical stiffness to the application.^13,14,37,38^ Hydrogel mechanics is an important property to consider for biomedical applications, as different locations in the body will require different abilities to withstand high stress or frequent movement.^40^ The range of mechanical properties exhibited by tissues in the body ranges from 1 kPa up to 1 MPa (Fig. 1), demonstrating the diverse needs of tissue replacement. It is desirable to have predictive control of hydrogel mechanics to replicate any given tissue stiffness, for example to include applications in skin tissue replacement for wound healing or cardiac muscle tissue for treating heart diesease.^41,42^

Limited studies have utilized folded protein hydrogels for the delivery of drugs, with the current focus including delivering growth factors.^43^ A major current challenge for protein hydrogel systems is the ability to control the triggered release of drug molecules from these hydrogels. Typically, drug release from protein hydrogels relies on diffusion of the drug out of the hydrogel. This has limitations of short timescales for drug release and a lack of control to tune the kinetics of drug release to suit different applications, leading to a need for repeated administration.^24^ Incorporating a triggered release mechanism would allow for the release of drugs at the desired time point, increasing the window for efficacy and potentially decreasing the side effects of the drug.^44–46^

**Fig. 2:**
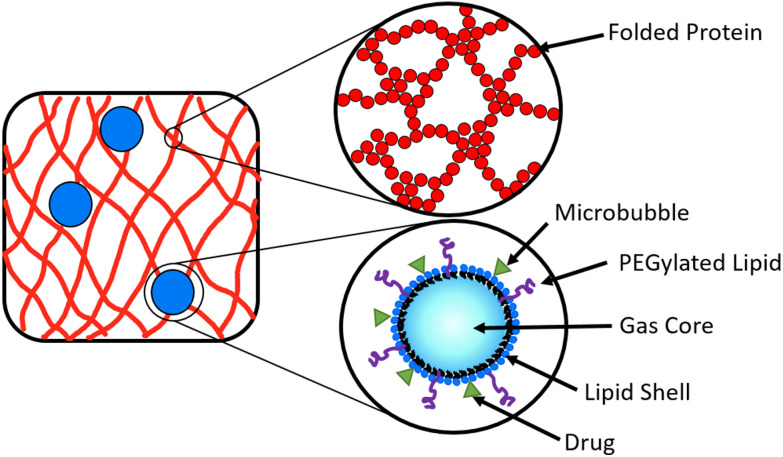
Schematic depicting microbubbles (blue spheres) encompassed within a folded protein hydrogel network (red lines), where the hydrogel network is composed of cross-linked folded proteins (red spheres). The inset of the MB shows drug molecules (green triangle) attached to the lipid shell, where the lipid shell stabilises the gas core.

A potential route to achieve controlled drug delivery is through the use of microbubbles (MBs), composed of a gaseous core stabilised by a thin shell and with diameters of 1–10 μm. MBs can be loaded with drugs, either by including drugs in the MB shell (Fig. 2) or in liposomes attached to the MB shell.^47–49^ Ultrasound (US) exposure of MBs instigates volumetric oscillations of the MBs, and by increasing the US amplitude, MBs can be burst to release loaded drugs on demand.^50–53^ Drug-loaded MBs, embedded within a hydrogel, provide a potential opportunity to impede the diffusion of drug molecules in the hydrogel and prolong the release profile. The time of drug release from the MBs into the surrounding hydrogel network can be controlled with the application of US which instigates the destruction of MBs.^54–57^ In a system of MBs undergoing US induced oscillations in a colloidal gel, the microstructure of the gel is influenced by the microstreaming from MBs.^58^ To date, MBs have not been embedded in folded protein hydrogels. Such a multi-composite system provides an opportunity to exploit the bursting of MBs to modulate the hydrogel porosity and small molecule or loaded drug diffusion, which could be controlled by the concentration of MBs.^59,60^

In previous studies of hyaluronic acid–carboxymethylcellulose hydrogels with embedded MBs, the release of fluorescently tagged dextran was controlled by changing either the concentration of MBs, the duration of the US pulse or the US amplitude.^61^ This approach has provided a solution to control the release of dextran and basic fibroblast growth factor from fibrous protein hydrogels^62,63^ and heparin from poloxamer 407 hydrogels,^64^ but has not yet been applied to hydrogels made from globular folded proteins that maintain and exploit the protein function. Using folded protein hydrogels has the potential to provide a biologically functional hydrogel scaffold that complements drug delivery, improving the overall efficacy of treatment. Furthermore, by encompassing drug-loaded MBs in protein hydrogels, the drug release profile could be controlled.

In previous studies on cellulose nanofibrils based gel, the inclusion of silica nanoparticles was shown to have a significant impact on the mechanical properties of the gel.^65^ Interestingly the relative size of nanoparticle to network mesh size was important. For example, a ×170 increase in storage modulus (*G*′) was observed when nanoparticles were greater than the network mesh size, while a ×60 increase in *G*′ was observed with nanoparticles less than the mesh size. Similarly, the inclusion of emulsion droplets of increasing volume fraction into gelatin hydrogels resulted in an increase in *G*′ of ×4.^66^ The inclusion of small unilamellar vesicles (SUVs) of 25 nm into gelatin hydrogels was compared to the inclusion of multilamellar vesicles (MLVs) of 5 μm in size. Both the addition of SUVs and MLVs led to a reduction in the Young's modulus of the gelatin, of 13.8% and 15.2% repsectively.^67^ A theoretical study of nanoparticles in polymer networks, where the nanoparticle underwent attractive interactions with the network, demonstrated that the size and volume fraction of the nanoparticle relative to the network building block alters the mesoscale structure of the network.^68^ Furthermore, experimental and modelling studies have shown that the so-called ‘filler’ particle size, volume fraction and interaction between the particle and the network can influence the bulk properties of the network.^69,70^

In the present study, we explore how inclusion of embedded MBs impacts the mechanical and structural properties of folded protein hydrogels. The MBs will act as micrometre sized filler particles within the cross-linked hydrogel network. The presence of the MBs may affect the network formation, since the stiffness of the MBs (∼1 GPa) is greater than the stiffness of BSA hydrogels (3 kPa at a volume fraction of 7%), and the size of MBs (1–10 μm) is much greater than the size of a folded BSA protein (3.5 nm).^14,71,72^

We have studied phospholipid stabilised MBs encompassed within chemically cross-linked globular BSA protein hydrogels (BSA : MB hydrogels). We use this as a model system to examine the stability and diffusion of the MBs within the hydrogel, the nanoscale and mesoscale properties of the protein network and the viscoelastic properties of the composite MB–hydrogel system. We present rheological characterization to identify the mechanical properties of these MB–hydrogel systems. We investigated the structure with small angle neutron scattering (SANS). BSA is a suitable protein for hydrogel formation, due to exposed tyrosine residues that photo-chemically cross-link in the presence of NaPS and Ru(BiPy)_3_.^73^ We have tested for the folded state of BSA post-gelation in the presence of MBs with circular dichroism (CD) spectroscopy, as well as studied the stability and diffusion of MBs with optical microscopy.

## Experimental

### Materials

Lipids 1,2-dipalmitoyl-*sn-glycero*-3-phosphocholine (DPPC), 1,2-distearoyl-*sn-glycero*-3-phosphoethanolamine-*N*-[amino (polyethylene glycol)-2000] (DSPE-PEG-2000) and atto-647n-1,2-dioleoyl-*sn-glycero*-3-phosphoethanolamine (atto-647n-DOPE) were purchased from Avanti Polar Lipids (Alabaster, AL, USA). Bovine serum albumin (BSA), sodium persulfate (NaPS), tris(2,2′-bipyridyl) dichlororuthenium(ii) hexahydrate (Ru(BiPy)_3_), glycerol, methanol, chloroform and silicone oil were purchased from Sigma-Aldrich Co. Ltd (Dorset, U.K.). Perfluorobutane (C_4_F_10_) was purchased from F2 Chemicals (Preston, UK).

### Microbubble production and characterisation

MBs were prepared from DPPC and DSPE-PEG-2000 mixed in 95 : 5 mol% ratio to give a final lipid concentration of 5 mg mL^−1^. For fluorescent MBs, additional lipid atto-647n-DOPE was added at 0.1 mol%. All lipids were dissolved in a 1 : 1 mixture of methanol and chloroform. To remove the solvent, the lipid mixture was dried under nitrogen for 1 hour and stored overnight under vacuum. The dried lipid film was resuspended in sodium phosphate (25 mM, pH 7.4) and 1% (v/v) glycerol, heated to 60 °C, followed by tip sonicating the lipid solution for 30 min to promote resuspension. The lipid solution was centrifuged at 17 000*g* to remove remnants of the tip, and the resultant supernatant used for producing MBs. C_4_F_10_ was used to saturate the lipid solution before shaking with ESPE CapMix (3M, USA) to produce MBs. To separate the larger MBs (>3 μm) from the solution, the MBs are stored at 4 °C for 30 min, such that larger, more buoyant MBs rise to the top of the centrifuge tube according to the Hadamard–Rybczynski equation.^74^ Optical imaging was used to determine the size and concentration of the MBs with an inverted microscope (Nikon, Japan) using a 40× magnification objective, as previously described.^75^ The captured images were analysed with a custom MATLAB (MathWorks, US) script using the Image Analysis Toolbox.^76^

Fluorescent images of MBs with fluorescently tagged Atto-647n-DOPE lipids were imaged in the BSA : MB hydrogel with a Leica DMi8/SP8 confocal microscope. The samples were excited using a 638 nm diode laser. Fluorescence emission from Atto-647n-DOPE lipid was collected from 590–768 nm. The confocal pinhole size was 1 A.U and a 50× objective was used.

### Preparation of BSA hydrogel in the absence and presence of microbubbles

BSA protein was resuspended at 200 mg mL^−1^ in sodium phosphate (25 mM, pH 7.4) and 1% (v/v) glycerol. The BSA solution was centrifuged for 1 min at 5000*g* and the supernatant used. NaPS and Ru(BiPy)_3_ required for the photochemical cross-linking reaction were mixed at 150 mM and 0.3 mM before storing at −80 °C until use. The Ru(BiPy)_3_ and NaPS were diluted with buffer for standard BSA hydrogels or MB solution for BSA : MB hydrogels. The Ru(BiPy)_3_/NaPS/buffer solution or the Ru(BiPy)_3_/NaPS/MB solution was mixed with BSA such that the final concentrations in the pre-gel solution were 100 mg mL^−1^ BSA, 50 mM NaPS, 0.1 mM Ru(BiPy)_3_. The pre-gel solution was irradiated with a blue LED light (452 nm) at *I* = 0.48 A for 5 min to form the hydrogel.

### Stability assessment of microbubbles

MBs in buffer and MBs in the BSA hydrogel were prepared and stored at 4 °C. Optical microscopy was used to compare the concentration of MBs in the two systems over a 48 h period. The concentration of MBs stored in buffer was assessed as previously described for MB characterisation. The concentration of MBs in the BSA : MB hydrogel was monitored by taking transmitted bright field images with the Leica DMi8/SP8 confocal microscope, using a 488 nm OPSL laser. *Z*-stacks of the BSA : MB hydrogels compiled of 50 images taken in 1 μm steps. Images were analysed with ImageJ and MosiacSuite plug-into determine the concentration of MBs.^77^

### Ultrasound set-up for the destruction of microbubbles

US exposure was used to destruct the MBs. A 2.25 MHz centre frequency unfocused transducer (V323-SM, Olympus, US) was used to deliver the US pulse, generated by a function signal generator (TG5011, Agilent Technologies, UK). The signal was amplified by a +53 dB RF power amplifier (A150, Electronics % Innovation, US). The US pulse repetition frequency was 1 kHz, with a 1% duty cycle for 4 s. The peak negative pressure was 900 kPa and the mechanical index was 0.6. The transducer was coupled to the glass coverslip with coupling gel and a gel pad (Aquaflex, Parker Laboratories, US).

### Rheology of protein hydrogels

To characterize the mechanical properties of the viscoelastic BSA and BSA : MB hydrogels, an Anton Paar MCR 502 stress-controlled rheometer (Anton Paar GmbH, Austria) was used, with a parallel plate geometry (plate diameter = 8 mm). The final concentration of BSA : MB hydrogels for rheological characterisation was 10^9^ MB per mL. The sample was loaded onto the rheometer such that the gap height was 0.85 mm. A thin layer silicone oil (viscosity = 5 ct) was applied to prevent sample evaporation during the measurements. The viscosity of the silicone oil was too low to effect the results, due to the torque range of the rheometer. Time sweep experiments were carried out at a frequency of 1 Hz, a constant shear strain (*γ*) of 0.5%, to ensure the samples were measured in the linear viscoelastic regime. After 1 min, the blue LED light was turned on for a further 5 min, initiating gelation. All measurement were performed at 22 °C, and the storage and loss (*G*′′) moduli were recorded for 65 min, to observe the viscoelastic properties for 1 hour after the LED light is turned off. The gelation curve was fitted with an empirical formula:^13^1
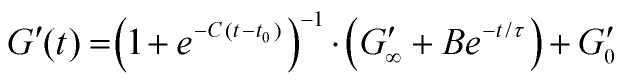
where *C* is related to the rate of increase of *G*′ after the photo-chemical cross-linking has started, *t*_0_ is the midpoint of the rate of increase of *G*′, *G*′_∞_ is the relaxed storage modulus at *t* = ∞ s, *B* is the coefficient of relaxation, *τ* is the time constant of relaxation and *G*_0_ is the *G*′ before photo-initiation. The BSA and BSA : MB hydrogels were then observed for a range of frequencies, 0.01–10 Hz and constant *γ* = 0.5%. From this the loss ratio, tan(*δ*), was determined from tan(*δ*) = *G*′′/*G*′. Stress–strain curves were obtained by loading and unloading at a strain rate of 1% s^−1^ up to 10, 30 and finally 50%, with 5 min of relaxation between each load/unloading cycle. The non-linear behaviour of the hydrogel was investigated at a frequency of 1 Hz, by increasing the shear strain logarithmically from 1–1000%.

### Circular dichroism

Circular dichroism (CD) experiments were performed on a Chirascan plus circular dichroism 719 spectrometer (Applied PhotoPhysics) and used to determine the secondary structure of BSA and BSA : MB hydrogels. Samples were loaded into a cuvette with a path length of 10 μm at 25 °C. The spectra were measured in the wavelength range 178–260 nm in steps of 1 nm, with a bandwidth of 5 nm as described previously.^13,14^

### Small angle neutron scattering

Small angle neutron scattering (SANS) experiments were performed at ISIS Neutron and Muon Source (STFC Rutherford Appleton Laboratory, Didcot, UK) using time-of-flight instrument Sans2d with the rear detector at 12 m and the front detector at 5 m from the sample, giving a wavenumber range of 0.0015 ≤ *q* ≤ 1 Å^−1^. The pre-gel solutions in 98% D_2_O (the lipid preparation in H_2_O buffer) were loaded into quartz cuvettes with 1 mm path length. Experiments were executed at 20 °C, controlled by a thermal bath. The scattering from an empty quartz cuvette was measured and used for accurate buffer subtraction of the sample. The raw data was reduced and corrected for transmission and detector efficiencies, and normalised on an absolute scale with scattering from a partially deuterated polystyrene standard using the Mantid framework (https://www.mantidproject.org). The resultant, absolute scattering curves were fitted with SASview (https://www.sasview.org). To fit a folded protein hydrogel, eqn (2) was used from previous studies,^13,14^ for the BSA hydrogels without MBs:

2 *I*(*q*) = *ϕ*_p_*V*_p_Δ*ρ*_p_^2^*P*_p_(*q*)·[(1 − *p*_c_) + *p*_c_*S*(*q*)] + *bkd*

where *ϕ*_p_ is the volume fraction of the protein, *V*_p_ is the volume of the protein, Δ*ρ*_p_ is the difference in contrast between the protein and the buffer, *P*_p_(*q*) is the ellipsoidal form factor of the building block,^78^*p*_c_ is the amount of protein in clusters, and *S*(*q*) is the fractal structure factor:^79,80^3
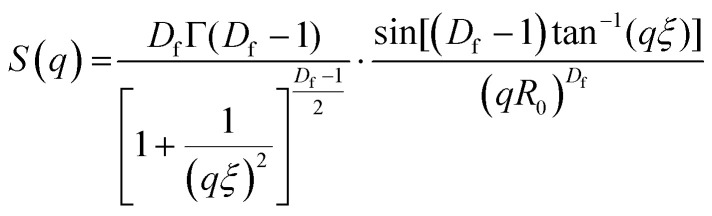
where *D*_f_ is the fractal dimension and *ξ* is the correlation length. Due to the use of DSPE-PEG-2000 in the MB shell, we expect that there will be no significant interactions between the MBs and the BSA hydrogel network that would affect the structure. Therefore, we can add an additional spherical form factor term, *P*_b_(*q*), to the model as described in eqn (4):4
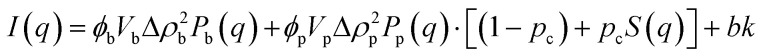


The radial distribution function, *g*(*r*), in eqn (5) derived by Teixeiria was used to determine the structure factor.^60^ In an extended analysis of structural data for folded protein hydrogels, eqn (5) was previously used to determine the number of protein building blocks in a cluster, *N*(*r*):^13,14^5
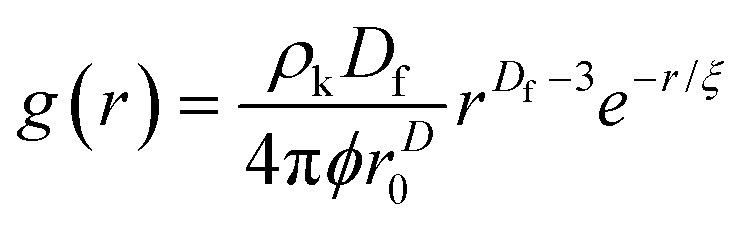
6
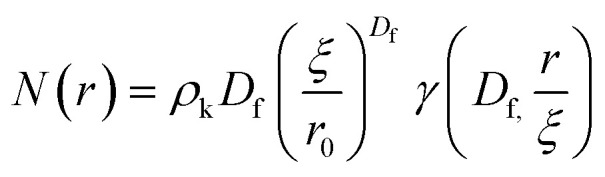
where *ρ*_k_ is the maximum packing density of the system, *r*_0_ is the minimum radius cut-off in this case the radius of the protein building block and 
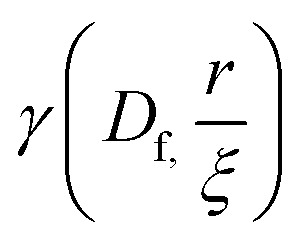
 is the lower incomplete gamma function.

## Results

### Optical observations of microbubbles in BSA hydrogels

**Fig. 3:**
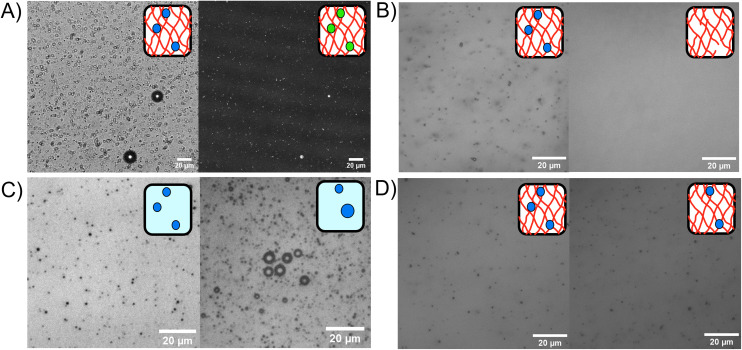
(A) Left: bright field microscopy of MBs in a cross-linked BSA hydrogel. Right: atto-647n fluorescent emission from fluorescently tagged MB lipid shell in a BSA hydrogel with confocal microscopy. (B) Left: MBs in a BSA protein hydrogel before ultrasound, Right: and after ultrasound destruction. (C) Microbubbles in buffer solution. Left: 1 h after MB production, 100× diluted. Right: 48 h after production, 10× dilution. (D) Microbubbles in BSA hydrogel. Left: 1 h after MB production. Right: 48 h after production. Cartoon schematics depict the presence and absence of MBs.

Phospholipid stabilised MBs were embedded within the BSA hydrogel, as shown in the bright field and fluorescent confocal microscopy images in Fig. 3A, where the MB concentration was 1 × 10^9^ MB per mL with an average size of 1.2 μm. An example MB size distribution is shown in ESI Fig. 1.†Fig. 3B–D show the MBs in bright field with inverted microscopy. Fig. 3B shows MBs dispersed in the hydrogel at a concentration of 3.0 × 10^10^ MB per mL before and after the application of US, where the resultant concentration after US was 2.6 × 10^8^ MB per mL. The 99.1% reduction of MBs after US exposure demonstrates the successful destruction MBs upon application of US while using a clinically safe mechanical index (MI) of less than 0.7.^81^ The MI quantifies the acoustic pressure and applied frequency, where high MI of over 1.9 will result in induced cavitation and unsafe levels of heating to tissue.^82^

The stability of the MBs stored in a fridge at 4 °C was determined by monitoring the presence of MBs in the BSA hydrogel over a 48 hours period. Fig. 3C shows MBs in solution at 0 hours (100× dilution) and 48 hours (10× dilution) post-production. ESI Fig. 1† shows microbubble size distributions for triplicate experiments at 0 hours and 48 hours. For comparison, the MBs stored in the hydrogel at 0 and 48 hours are shown in Fig. 3D. In Fig. 3C, the concentration of MBs at 0 hours was 2.6 ± 0.2 × 10^10^ MB per mL, and at 48 hours later was 2.9 ± 1.1 × 10^9^ MB per mL, with an 89% reduction in MB population in solution after 48 h. Fig. 3D shows MBs in the BSA hydrogel at 0 hours, at a concentration of 1.22 ± 0.04 × 10^10^ MB per mL and 48 hours post-production, where the concentration was 7 ± 1 × 10^9^, showing a 39% reduction in the concentration of MBs after 48 hours in the BSA hydrogel (ESI Fig. 2†). The larger reduction in MBs observed when storing the MBs in buffer solution, demonstrates that the MBs are less stable when stored in the buffer solution compared to storage in the BSA hydrogel. The increased stability of MBs in the hydrogels may be attributed to the prevention of coalescence and the inability of MBs to float to an air interface, as the MBs are restricted in position by the surrounding covalently cross-linked network.^83^ ESI Fig. 3† shows the trajectories of MBs in buffer solution compared to the MBs trapped in the BSA hydrogel, measured with optical microscopy to track the movement of the MBs. The MBs diffuse freely in buffer solution (diffusion coefficient of 3 ± 2 × 10^−11^ m^2^ s^−1^ in the *X* direction), while their diffusion is significantly reduced to 7 ± 2 × 10^−13^ m^2^ s^−1^ when embedded in the protein hydrogel, showing that the hydrogels are an effective scaffold for reducing the movement and subsequent coalescence of MBs.

### Assessing the nanoscale structure of the protein building block within the protein network

**Fig. 4:**
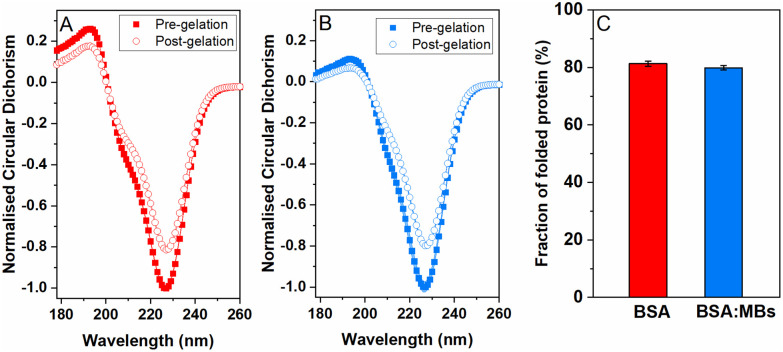
Normalized circular dichroism spectra of BSA for (A) the standard BSA gel and (B) BSA : MB gel at a MB concentration of 10^9^ MB per mL, with spectra included for both before gelation (squares) and post-gelation (circles). (C) Comparison of the peak at 222 nm associated with the α-helical structure of BSA.

To quantify the amount of folded protein in the BSA and BSA : MB hydrogels, CD was used to measure the signature of BSA secondary structure in the pre-gel solution and post-gel cross-linked network. Protein unfolding during network formation and relaxation defines the structure and mechanical properties of folded protein hydrogels.^14,32^ Here, we determine the impact of embedded MBs on the proportion of folded protein in the cross-linked protein network.^23,25^ BSA has a purely α-helical structure, with well characterised negative peaks at 222 nm and 209 nm.^84,85^Fig. 4 shows the normalised CD spectra from the BSA hydrogels (Fig. 4A) and the BSA : MB hydrogels (Fig. 4B) both pre- and post-gelation. Both spectra show a purely α-helical structure, with well-characterised negative peaks at 222 nm and 209 nm. The slight shift in spectra from the expected peaks can be attributed to the high protein concentration used, resulting in a high signal to noise ratio spectra. Fig. 4C compares the folded fraction of BSA post-gelation at the 222 nm peak, for the BSA hydrogels (81.3 ± 0.9%) and the BSA : MB hydrogels (79.9 ± 0.8%). As expected, the majority of BSA remains folded after gelation due to the presence of 17 intramolecular disulfide bonds, that act as strong covalent staples within BSA, creating a high energy barrier to unfold BSA.^14^ Comparing the folded fraction of BSA protein with and without MBs, post-gelation, showed no significant change, with the folded fractions being within error.

### Linear and non-linear mechanical properties of BSA hydrogel with embedded microbubbles

**Fig. 5:**
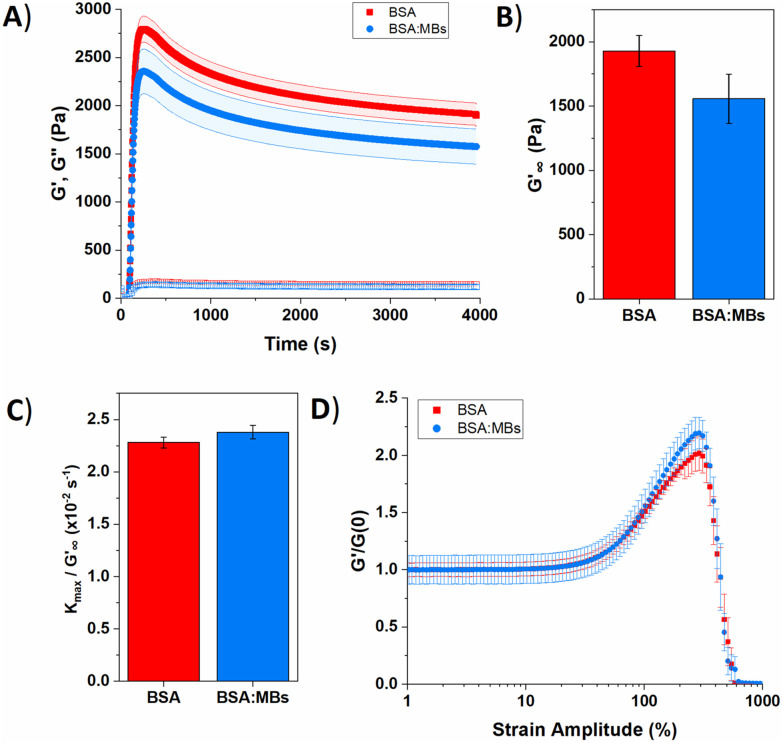
(A) Gelation curve show the storage (filled) and loss moduli (hollow) over time for control BSA hydrogel in the absence (red squares) and presence (blue circles) of MBs. (B) Relaxed storage modulus (*G*′_∞_) from fitting eqn (1) and (C) kinetic parameters, *k*_max_, normalized to *G*′_∞_ with changing the MB concentration. (D) Non-linear behaviour of BSA hydrogels in the absence (red squares) and presence (blue circles) of MBs.

We measured the viscoelastic properties of BSA protein hydrogels of both BSA and BSA : MB hydrogels (10^9^ MB per mL) using shear rheology. Fig. 5A shows the gelation curve for BSA and BSA : MB hydrogels, characterised by the elastic, *G*′, and viscous, *G*′′, moduli. The photo-initiated chemical crosslinking was enabled by switching on an LED lamp 60 s after loading the sample, to initiate the chemical cross-linking reaction between BSA proteins. The lamp was left on for 300 s to ensure complete dityrosine bond formation and for both hydrogels, *G*′ rapidly increases, becoming larger than *G*′′, indicating the viscoelastic liquid is becoming a viscoelastic gel. After 300 s of photo-initiated chemical cross-linking, *G*′ reaches a maximum peak and then decreases, indicating relaxation of the protein network. Network relaxation was monitored for one hour after the lamp is switched off.

Several parameters of interest can be extracted from the gelation curves shown in Fig. 5A. Firstly, by fitting the gelation curves with eqn (1), the relaxed storage modulus (*G*′_∞_), defined as the *G*′ at time *t* = ∞, can be determined.^13,14^ An example of the fittings is shown in ESI Fig. 4.†Fig. 5B shows that when comparing *G*′_∞_, a reduction in the stiffness of the relaxed BSA : MB hydrogel (1557 ± 190 Pa) relative to the BSA hydrogel (1928 ± 120 Pa) is observed. The relaxation time constant (*τ*) was also extracted, again using eqn (1), shown in ESI Fig. 5† to have an minor increase in *τ* for BSA : MB hydrogel (1163 ± 17 s) compared to the BSA hydrogel (1145 ± 13 s). The kinetics of gelation is extracted from Fig. 5A, determined by the maximum derivative, *k*_max_, of the linear region that occurs immediately after photo-initiation. The *k*_max_ describes the rate of gelation as a result of the photo-chemical cross-linking reaction and is compared for the BSA and BSA : MB hydrogels. An example of the fitting is shown in ESI Fig. 6.†Fig. 5C shows *k*_max_ with respect to *G*′_∞_. The addition of MBs to the BSA hydrogel results in a 4 ± 1% change in *k*_max_/*G*′. This suggests that at the concentration used in this study with 10^9^ MB per mL, the MBs are not significantly impeding the rate of chemical crosslinking during network formation. Such subtle changes in hydrogel mechanics might be expected given the low volume fraction of MBs (1%) in the composite system. To quantify the degree of chemical cross-linking in the BSA and BSA : MB hydrogels, the fluorescence emission of dityrosine was measured in the BSA hydrogel in the presence and absence of 10^9^ MB per mL. ESI Fig. 7† shows the fluorescence emission in the BSA hydrogel in the absence of MBs is 140 000 ± 20 000 and 160 000 ± 10 000 for the BSA : MB hydrogels. This suggests that the MBs are not significantly impeding the number of chemical cross-links between BSA proteins during network formation. The fluorescence assay shows that the number of crosslinks in each system is similar, which might imply that the storage modulus of the two systems are identical. However, the mechanical measurements show a reduction in *G*′ suggesting a mesoscale structural change to the arrangement of the cross-linked proteins.

The gelation curves (Fig. 5a) show the evolution of the shear storage modulus (*G*′) during the formation of BSA hydrogels in the absence and presence of MBs. The gelation curves show the expected profile of an initial sharp increase in *G*′, due to photoactivated chemical cross-linking, followed by slow relaxation. This profile is consistent with previous studies of chemically crosslinked BSA hydrogels and maltose binding protein (MBP) hydrogels.^13,86^ The rheology curves can be fit with an equation to extract information on the *G*′ relaxation of the system. In the case of MBP hydrogels two modes of relaxation are measured; a fast relaxation which is the rearrangement of the percolated hydrogel network, and a second, slower relaxation which is the unfolding of the protein building block.^86^ Further, previous work on BSA in the absence of a reducing agent (DTT) displays one relaxation mode, while in the presence of DTT the disulphide bonds in the BSA are broken by the reducing agent and two relaxation modes are measured.^14^ Combined with additional experiments which use CD to probe secondary structure content in proteins, this suggests that the emergence of two-relaxation modes is inherently linked to force lability of the protein during gelation.

In the present study, the gelation curve in Fig. 5A was fitted with both one and two exponential decay constants. The results showed that the gelation curve was best fitted with one exponential decay term (see ESI Fig. 4†). This is consistent with a protein hydrogel network in which the majority of the proteins remain folded, as observed in the nanoscale protein structure data in Fig. 4A and B. A frequency sweep provided insight into the post-gelation viscoelastic properties of BSA protein hydrogels both with 10^9^ MB per mL and without MBs. We observe that *G*′ is greater than *G*′′ in both the presence and absence of MBs (ESI Fig. 8†). *G*′ was greater by 300 Pa for the BSA hydrogel in the absence of MBs, consistent with Fig. 5A.

The BSA hydrogels were loaded and unloaded with an applied shear strain to understand their response to force, mimicking the repeated force that hydrogels may experience in biomedical applications.^41^ ESI Fig. 9A† shows a linear relationship between the shear strain and shear stress occurs up to a shear strain of 20%. At a shear strain of 50%, the shear stress for the BSA hydrogels was only slightly reduced with the addition of MBs. The energy dissipation of the BSA hydrogel showed a 20% decrease with the addition of 10^9^ MB per mL to the BSA hydrogel, suggesting the inclusion of MBs does not severely impact the ability of the hydrogel to recover from an applied stress (ESI eqn (1) and ESI Fig. 9C†).

The non-linear behaviour of hydrogels lends itself to biomedical applications, where for example a hydrogel biomaterial will undergo high strain deformation from movement of the body.^87^Fig. 5D shows the non-linear behaviour of BSA and BSA : MB hydrogels, normalized to the *G*′ before photo-initiation, *G*′(0). For both the BSA and BSA : MB hydrogels, linear deformation occurs up to a strain amplitude of 30%, after which the hydrogels shear, stiffen and eventually break. BSA : MB hydrogels exhibit an increased strain stiffening from 50%. This behaviour may be due to the MBs supporting the network at high strain, as the stiffness of the MBs are significantly greater than the stiffness of the surrounding BSA network (∼1 GPa). Both BSA and BSA : MB hydrogels start to break at a strain amplitude of 290%. This suggests that the presence of MBs (10^9^ MB per mL) does not significantly weaken the BSA hydrogel.

The subtle reduction in stiffness of the BSA hydrogel with embedded MBs could be attributed to properties of the MBs. Studies investigating filler particles in soft materials suggest that a decrease, or little change, in material stiffness, with the inclusion of filler particles could be due to the filler particle not interacting with the network, or the filler particles at a low volume fraction, resulting in no measurable effect.^70,88^ In the composite system investigated here the mechanical changes are small, which may be explained by the low volume fraction of MBs included in the BSA hydrogels (approximately 1%). Similar changes were observed with the inclusion of GUVs in gelatin gels; at low volume fraction of 1.5%, a reduction in stiffness of 8.8% was measured.^67^ In the present study, the MBs contain DPPC lipids with additional PEGylated lipids. PEG molecules are formulated into many drug delivery systems, and improve the stability and lifetime of MBs and liposomes by reducing the removal rate from the blood stream *via* the mononuclear phagocyte system.^89–91^ The hydrophilicity and flexible chains of low molecular weight PEG molecules such as PEG-2000 have been shown, in both theoretical and experimental studies, to reduce serum albumin protein interaction with lipids.^92^ The MBs are therefore presumed to be not bound to the BSA hydrogel network. However, the presence of MBs may still lead to changes in the surrounding arrangement of BSA protein network. This is suggested by a simple thermodynamic expression of the shear modulus, *G*, of a cross-linked network:^93,94^7
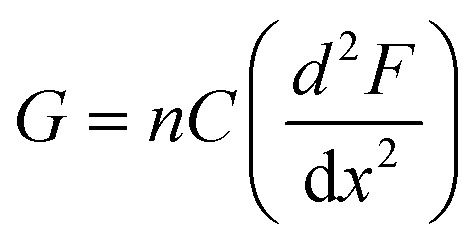
where *n* is the number of bonds per unit area, *F* is the Gibbs free energy, *x* is the cross-link length, *C* describes the structure of the network and how the mesoscale protein clusters are linked together, which leads to our structural investigation of BSA and BSA : MB hydrogels with SANS.

### Mesoscale structure of protein network with embedded microbubbles

**Fig. 6:**
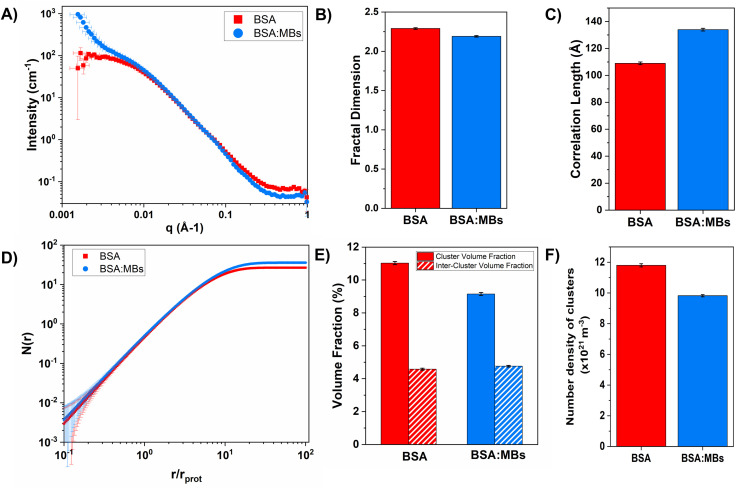
(A) SANS curves in the absence (red squares) and presence (blue circles) of MBs. (B) The fractal dimension and (C) correlation length predicted from the SANS curve, in the absence and presence of MBs. (D) Average number proteins in each cluster as a function of *r*, the distance from the centre of the cluster. (E) Volume fraction a cluster and inter-cluster space, and (F) the estimated number of clusters in the hydrogel for both in the absence and presence of MBs.

To determine the origin of the changes in the mechanical properties, the structure of the BSA and BSA : MB hydrogels was probed using SANS. SANS curves for BSA and BSA : MB hydrogels are shown in Fig. 6A. In the absence of MBs, the scattering curves show a similar profile to that previously measured for BSA hydrogels.^14,37^ In the low-*q* region the scattering curve shows an upturn in the BSA : MB hydrogel, which does not appear in the BSA hydrogel in the absence of MBs. The higher scattering intensity and inflexion at low-*q*, observed in the BSA : MB hydrogel curve, appears in the region where micron-sized objects such as MBs would be expected, confirming the presence of the MBs in the hydrogel. For MBs of size 1 μm, the plateau in the low-*q* region would be expected at *q* < 1.5 × 10^−4^ Å^−1^, therefore not in the detectable range of the instrument.

To investigate the structural changes we fitted our SANS data in order to extract key structural parameters. Previous studies have characterised the structural properties of folded globular proteins using SANS and SAXS and predicted a network with fractal-like clusters of globular folded protein, connected by an inter-cluster region populated by either folded or unfolded proteins.^13,14^ BSA-based hydrogels have been demonstrated to have fractal-like clusters of folded BSA connected by an inter-cluster region of folded BSA,^14^ therefore we use a similar fractal structure model to fit the SANS data presented in this work (eqn (2)–(4)). From fitting the SANS curves in Fig. 6A, the fractal structure factor is estimated and the fractal dimension (*D*_f_) and the correlation length (*ξ*) are extracted. *D*_f_ can be defined as a measure of how the mass of protein in a fractal cluster of proteins changes with volume, and intuitively can be thought of as related to the geometry and density of the cluster. *ξ* is the characteristic length scale of the fractal-like cluster with dimensionality, *D*_f_, relating directly to the upper limit of the fractal cluster *i.e.*, the size of the fractal protein clusters. The results in Fig. 6B and C show how *D*_f_ and *ξ*, respectively, change in the absence and presence of embedded MBs in the BSA hydrogel. Fig. 6B shows that *D*_f_ is slightly decreased upon the addition of MBs, where for BSA and BSA : MB hydrogels, *D*_f_ is 2.29 ± 0.01 and 2.19 ± 0.01. In contrast Fig. 6C shows that *ξ* increases when MBs are embedded in the gel network, where for BSA and BSA : MB hydrogels *ξ* is 109 ± 1 Å and 134 ± 1 Å. These results suggest that the size of the fractal-like clusters of proteins increases, without any significant change to the geometry or density of packing of the proteins in the clusters. Previous studies have shown that the *G*′ of a system composed of a network of clusters is not dependent on the size of the cluster, but on large changes in the volume fraction of the clusters and interactions between clusters.^95–97^ If this is the case for protein hydrogel systems, it suggests that the changes in *G*′ measured in Fig. 5B are not due to the change in cluster size observed in Fig. 6C.

To investigate further, an analysis used in previous studies was applied, which allows the approximate number of protein building blocks in a cluster, *N*(*r*), and the average volume fraction of the cluster, *ϕ*_C_, and inter-cluster, *ϕ*_IC_, regions to be estimated (eqn (5) and (6)).^13,37^Fig. 5E shows that network formation in BSA : MB hydrogels results in a lower *ϕ*_C_ (9.2 ± 0.1%) compared to the BSA hydrogel without MBs (11.0 ± 0.1%), while the *ϕ*_IC_ of the inter-cluster region shows an increase in BSA : MB hydrogels (4.76 ± 0.05%), compared to the BSA hydrogel without MBs (4.58 ± 0.06%). Previous studies on clustered colloidal networks show that changes to *ϕ*_C_ on order of 2% observed in this study do not significantly impact the mechanical properties of the hydrogel network, but require changes of upward of 10% in *ϕ*_C_ for significant changes in the mechanics.^96^

Using eqn (6), the number of clusters in the hydrogel network can be calculated (Fig. 6F). In the presence of MBs, the number of clusters in the network reduces by ∼19%. Interestingly, this correlates with the measured reduction in *G*′ of approximately ∼20% in the presence of MBs (Fig. 5B). This may be explained by classic affine network theory, where the *G*′ of a network is linearly dependent on the number density of load-bearing chains in the network.^98^ If the number of chains is linearly related to the number of junction points, the *G*′ of the material will also be linearly dependent on the number density of the junction points. With less junction points and therefore connecting chains, the stiffness of the network will decrease. Applying this to BSA hydrogels, we propose that the clusters of proteins may act as junction points between the inter-cluster region of unfolded chains of BSA monomers. The observed reduction in *G*′ for the BSA : MB hydrogel (Fig. 5B) may result from the growth of the number of proteins in a cluster at the expense of the number of cluster junction points.^99^ Interestingly, previous work has shown that the mechanical properties and the fracture of disordered collagen networks is controlled by the connectivity of the fibres. The collagen network's fracture strain is controlled by the coordination number of the network junctions, with less connected networks fracturing at larger strains.^99^ This, along with the results in this study, suggests the number of network junctions is important in determining the mechanical properties of a folded protein hydrogel network. Future studies to manipulate the protein cross-linking and density of network junction will reveal the potential for tuning the protein hydrogel properties for a range of biomedical applications.

## Conclusions

**Fig. 7:**
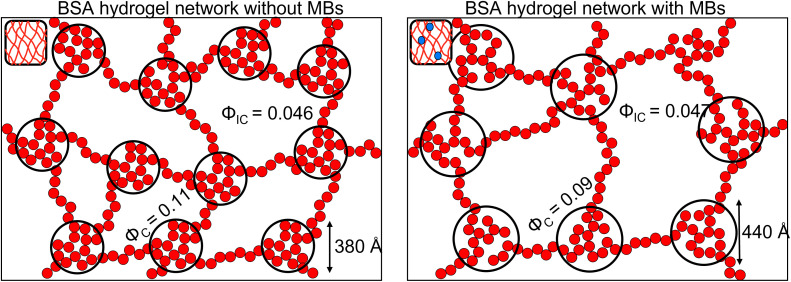
Predicted structure of the BSA hydrogel network Left in the absence of MBs and Right in the presence of MBs. The smaller red and blue circles represent folded proteins, the black circles represent the fractal-like clusters (*ϕ*_C_) connected with proteins in the inter-cluster space (*ϕ*_IC_).

We have presented a novel folded protein hydrogel based material with embedded MBs. We quantify the changes to the mechanical properties of the BSA hydrogel and find a 300 Pa reduction in *G*′. This allows a high concentration of MBs (10^9^ MB per mL) to be embedded within protein hydrogels without concerns about weakening the hydrogel stiffness. We characterise the nanoscale and mesoscale structure of the protein hydrogel. At the nanoscale, the folded fraction of the BSA : MB hydrogel remains the same, providing opportunities for exploiting the biological functionality of the protein hydrogel building block. Given the protein fold is sensitive to mechanical and chemical cues, this provides future opportunities to create a responsive protein hydrogel, which can complement a composite drug delivery system. The mesoscale structural insight shows a decrease (19%) in the number of clusters in networks forming in the presence of MBs (Fig. 7). This suggests that protein clusters act as important junction points, which modulate the hydrogel network mechanics. The lifetime of MBs was significantly increased in the BSA hydrogel and their diffusion impeded, providing future opportunities for long-term storage and more complex release profiles of drug-loaded MBs. There are generally 3 mechanism of MB loss, (i) floatation and bursting, (ii) coalescence and (iii) dissolution.^100–102^ Embedding the MB within a hydrogel network prevents floatation and bursting and coalescence – removing two mechanism for MB loss. The 3rd mechanism, dissolution, is described by a modified Epstein Plessett relation and would be same for bubbles in solution or the gels.^102^ Thus our observed increase in MB stability is likely due to the fact that processes (i) and (ii) are hindered/prevented. Of these 2 processes floatation/bursting is likely to be the dominant loss mechanism over coalescence. MBs were successfully burst within clinically safe US parameters, allowing the next experimental steps to include embedding drug-loaded MBs and monitoring drug release. Further work to explore the impact of MB oscillations on the mesoscale structure of the protein network, as previously demonstrated in colloidal gel systems, could offer a route to understanding local mechanical properties in addition to the bulk mechanical properties investigated in this study.^58^

## Conflicts of interest

There are no conflicts to declare.

## Supplementary Material

BM-011-D2BM01918C-s001

## References

[cit1] Kellermayer M. S. Z., Bustamante C., Granzier H. L. (2003). Biochim. Biophys. Acta, Bioenerg..

[cit2] Puchner E. M., Gaub H. E. (2009). Curr. Opin. Struct. Biol..

[cit3] Higham T. (2011). Science.

[cit4] Marinelli J. P., Levin D. L., Vassallo R., Carter R. E., Hubmayr R. D., Ehman R. L., McGee K. P. (2017). Magn. Reson. Imaging.

[cit5] Murphy M. C., Jones D. T., Jack C. R., Glaser K. J., Senjem M. L., Manduca A., Felmlee J. P., Carter R. E., Ehman R. L., Huston J. (2016). NeuroImage Clin..

[cit6] Kolipaka A., Wassenaar P. A., Cha S., Marashdeh W. M., Mo X., Kalra P., Gans B., Raterman B., Bourekas E. (2018). Clin. Imaging.

[cit7] Samir A. E., Allegretti A. S., Zhu Q., Dhyani M., Anvari A., Sullivan D. A., Trottier C. A., Dougherty S., Williams W. W., Babitt J. L., Wenger J., Thadhani R. I., Lin H. Y. (2015). BMC Nephrol..

[cit8] Park S., Tao J., Sun L., Fan C. M., Chen Y. (2019). Molecules.

[cit9] Eby S. F., Cloud B. A., Brandenburg J. E., Giambini H., Song P., Chen S., Lebrasseur N. K., An K. N. (2015). Clin. Biomech..

[cit10] Stefanescu H., Grigorescu M., Lupsor M., Procopet B., Maniu A., Badea R. (2011). J. Gastroenterol. Hepatol..

[cit11] Nenadic I., Mynderse L., Husmann D., Mehrmohammadi M., Bayat M., Singh A., Denis M., Urban M., Alizad A., Fatemi M. (2016). PLoS One.

[cit12] Robinson D. L., Kersh M. E., Walsh N. C., Ackland D. C., de Steiger R. N., Pandy M. G. (2016). J. Mech. Behav. Biomed. Mater..

[cit13] Hughes M. D. G., Cussons S., Mahmoudi N., Brockwell D. J., Dougan L. (2020). Soft Matter.

[cit14] Hughes M. D. G., Hanson B. S., Cussons S., Mahmoudi N., Brockwell D. J., Dougan L. (2021). ACS Nano.

[cit15] Gao X., Fang J., Xue B., Fu L., Li H. (2016). Biomacromolecules.

[cit16] da Silva M. A., Lenton S., Hughes M., Brockwell D. J., Dougan L. (2017). Biomacromolecules.

[cit17] Khoury L. R., Slawinski M., Collison D. R., Popa I. (2020). Sci. Adv..

[cit18] Shen Y., Levin A., Kamada A., Toprakcioglu Z., Rodriguez-Garcia M., Xu Y., Knowles T. P. J. (2021). ACS Nano.

[cit19] Silva N. H. C. S., Vilela C., Marrucho I. M., Freire C. S. R., Pascoal Neto C., Silvestre A. J. D. (2014). J. Mater. Chem. B.

[cit20] Tavakol D. N., Tratwal J., Bonini F., Genta M., Campos V., Burch P., Hoehnel S., Béduer A., Alessandrini M., Naveiras O., Braschler T. (2020). Biomaterials.

[cit21] Huang Y., Fitzpatrick V., Zheng N., Cheng R., Huang H., Ghezzi C., Kaplan D. L., Yang C. (2020). Adv. Healthcare Mater..

[cit22] Yang P., Song H., Qin Y., Huang P., Zhang C., Kong D., Wang W. (2018). Nano Lett..

[cit23] Sun W., Duan T., Cao Y., Li H. (2019). Biomacromolecules.

[cit24] Huerta-López C., Alegre-Cebollada J. (2021). Nanomaterials.

[cit25] Lei H., Dong L., Li Y., Zhang J., Chen H., Wu J., Zhang Y., Fan Q., Xue B., Qin M., Chen B., Cao Y., Wang W. (2020). Nat. Commun..

[cit26] Li Y., Xue B., Cao Y. (2020). ACS Macro Lett..

[cit27] Wang R., Fu L., Liu J. (2019). Chem. Commun..

[cit28] Fu L., Li H. (2020). Macromolecules.

[cit29] Wang Y., Li Z., Ouyang J., Karniadakis G. E. (2020). Soft Matter.

[cit30] Fu L., Haage A., Kong N., Tanentzapf G., Li H. (2019). Chem. Commun..

[cit31] Khoury L. R., Popa I. (2019). Nat. Commun..

[cit32] Hughes M. D. G., Cussons S., Mahmoudi N., Brockwell D. J., Dougan L. (2022). ACS Nano.

[cit33] Wu J., Li P., Dong C., Jiang H., Xue B., Gao X., Qin M., Wang W., Chen B., Cao Y. (2018). Nat. Commun..

[cit34] Lv S., Dudek D. M., Cao Y., Balamurali M. M., Gosline J., Li H. (2010). Nature.

[cit35] Kong N., Fu L., Peng Q., Li H. (2017). ACS Biomater. Sci. Eng..

[cit36] Bian Q., Fu L., Li H. (2022). Nat. Commun..

[cit37] Aufderhorst-Roberts A., Hughes M. D. G., Hare A., Head D. A., Kapur N., Brockwell D. J., Dougan L. (2020). Biomacromolecules.

[cit38] Fang J., Li H. (2012). Langmuir.

[cit39] Khoury L. R., Nowitzke J., Shmilovich K., Popa I. (2018). Macromolecules.

[cit40] Jacob S., Nair A. B., Shah J., Sreeharsha N., Gupta S., Shinu P. (2021). Pharmaceutics.

[cit41] Guimarães C. F., Gasperini L., Marques A. P., Reis R. L. (2020). Nat. Rev. Mater..

[cit42] Wu T., Liu W. (2022). NPG Asia Mater..

[cit43] King W. J., Mohammed J. S., Murphy W. L. (2009). Soft Matter.

[cit44] Hoarea T. R., Kohane D. S. (2008). Polymer.

[cit45] Tang J., Lee D., Tice T. R. (2022). Nat. Rev. Mater..

[cit46] Brandl F., Kastner F., Gschwind R. M., Blunk T., Teßmar J., Göpferich A. (2010). J. Controlled Release.

[cit47] Liu Y., Zhou Y., Xu J., Luo H., Zhu Y., Zeng X., Dong F., Wei Z., Yan F., Zheng H. (2021). Biomater. Sci..

[cit48] Bjånes T., Kotopoulis S., Murvold E. T., Kamčeva T., Gjertsen B. T., Gilja O. H., Schjøtt J., Riedel B., McCormack E. (2020). Pharmaceutics.

[cit49] Helfield B. (2019). Ultrasound Med. Biol..

[cit50] Ingram N., Mcveigh L. E., Abou-saleh R. H., Batchelor D. V. B., Loadman P. M., Mclaughlan J. R., Markham A. F., Evans S. D., Coletta P. L. (2022). Pharmaceutics.

[cit51] Charalambous A., Mico V., McVeigh L. E., Marston G., Ingram N., Volpato M., Peyman S. A., McLaughlan J. R., Wierzbicki A., Loadman P. M., Bushby R. J., Markham A. F., Evans S. D., Coletta P. L. (2021). Nanomedicine.

[cit52] Bourn M. D., Batchelor D. V. B., Ingram N., McLaughlan J. R., Coletta P. L., Evans S. D., Peyman S. A. (2020). J. Controlled Release.

[cit53] Luo T., Wang Z., He J., Hao L., Xiao L., Zhu Y., Wang Q., Pan X., Chang S. (2017). Cancer Lett..

[cit54] Olsman M., Sereti V., Mühlenpfordt M., Johnsen K. B., Andresen T. L., Urquhart A. J., Davies C. de L. (2021). Ultrasound Med. Biol..

[cit55] De Cock I., Zagato E., Braeckmans K., Luan Y., de Jong N., De Smedt S. C., Lentacker I. (2015). J. Controlled Release.

[cit56] Ingram N., McVeigh L. E., Abou-Saleh R. H., Maynard J., Peyman S. A., McLaughlan J. R., Fairclough M., Marston G., Valleley E. M. A., Jimenez-Macias J. L., Charalambous A., Townley W., Haddrick M., Wierzbicki A., Wright A., Volpato M., Simpson P. B., Treanor D. E., Thomson N. H., Loadman P. M., Bushby R. J., Johnson B. R. G., Jones P. F., Anthony Evans J., Freear S., Markham A. F., Evans S. D., Louise Coletta P. (2020). Theranostics.

[cit57] Kooiman K., Foppen-Harteveld M., van der Steen A. F. W., De Jong N. (2011). J. Controlled Release.

[cit58] Saint-Michel B., Petekidis G., Garbin V. (2022). Soft Matter.

[cit59] Lima E. G., Durney K. M., Sirsi S. R., Nover A. B., Ateshian G. A., Borden M. A., Hung C. T. (2012). Acta Biomater..

[cit60] Mauretti A., Neri A., Kossover O., Seliktar D., Di Nardo P., Melino S. (2016). Macromol. Biosci..

[cit61] Epstein-Barash H., Orbey G., Polat B. E., Ewoldt R. H., Feshitan J., Langer R., Borden M. A., Kohane D. S. (2010). Biomaterials.

[cit62] Moncion A., Arlotta K. J., O'Neill E. G., Lin M., Mohr L. A., Franceschi R. T., Kripfgans O. D., Putnam A. J., Fabiilli M. L. (2016). Acta Biomater..

[cit63] Fabiilli M. L., Wilson C. G., Padilla F., Martín-Saavedra F. M., Fowlkes J. B., Franceschi R. T. (2013). Acta Biomater..

[cit64] Lin T., Zhao X., Zhang Y., Lian H., Zhuang J., Zhang Q., Chen W., Wang W., Liu G., Guo S., Wu J., Hu Y., Guo H. (2016). Materials.

[cit65] Calabrese V., Da Silva M. A., Porcar L., Bryant S. J., Hossain K. M. Z., Scott J. L., Edler K. J. (2020). Soft Matter.

[cit66] Oliver L., Berndsen L., van Aken G. A., Scholten E. (2015). Food Hydrocolloids.

[cit67] Genot C. , Guillet S. and Metro B., Trends Colloid Interface Sci. III, 2007, pp. 18–23

[cit68] Sorichetti V., Hugouvieux V., Kob W. (2018). Macromolecules.

[cit69] Dickinson E., Chen J. (1999). J. Dispersion Sci. Technol..

[cit70] van Vliet T. (1988). Colloid Polym. Sci..

[cit71] Abou-Saleh R. H., Peyman S. A., Critchley K., Evans S. D., Thomson N. H. (2013). Langmuir.

[cit72] Axelsson I. (1978). J. Chromatogr. A.

[cit73] Fancy D. A., Kodadek T. (1999). Proc. Natl. Acad. Sci. U. S. A..

[cit74] Ruckenstein E. (2013). Colloids Surf., A.

[cit75] Armistead F. J., Batchelor D. V. B., Johnson B. R. G., Sally A., Evans S. D., Abou-saleh R. H. (2021). Rev. Sci. Instrum..

[cit76] Batchelor D. V. B. , 2020

[cit77] Sbalzarini I. F., Koumoutsakos P. (2005). J. Struct. Biol..

[cit78] Svergun D. I. and Geigin L. A., Structure Analysis by Small-Angle X-Ray and Neutron Scattering, Springer New York, NY, 1st edn., 1987

[cit79] Teixeira J. (1988). J. Appl. Crystallogr..

[cit80] Chen S. H., Teixeira J. (1986). Phys. Rev. Lett..

[cit81] ter Haar G. , in The safe use of ultrasound in medical diagnosis, ed. and ter Haar G., The British Institute of Radiology, 3rd edn, 2012, pp. 142–158

[cit82] USA Food & Drug Administration

[cit83] Borden M. A. (2019). Langmuir.

[cit84] Greenfield N. J. (2007). Nat. Protoc..

[cit85] Paul S., Sepay N., Sarkar S., Roy P., Dasgupta S., Sardar P. S., Majhi A. (2017). New J. Chem..

[cit86] Hughes M. D. G., Hanson B. S., Cussons S., Mahmoudi N., Brockwell D. J., Dougan L. (2021). ACS Nano..

[cit87] Kamkar M., Janmaleki M., Erfanian E., Sanati-Nezhad A., Sundararaj U. (2022). Can. J. Chem. Eng..

[cit88] Lewis T. B., Nielsen L. E., Company M. (1970). Appl. Polym. Sci..

[cit89] Munasinghe A., Mathavan A., Mathavan A., Lin P., Colina C. M. (2019). J. Phys. Chem. B.

[cit90] Shi D., Beasock D., Fessler A., Szebeni J., Ljubimova J. Y., Afonin K. A., Dobrovolskaia M. A. (2022). Adv. Drug Delivery Rev..

[cit91] Salmaso S., Caliceti P. (2013). J. Drug Delivery.

[cit92] Bartucci R., Pantusa M., Marsh D., Sportelli L. (2002). Biochim. Biophys. Acta.

[cit93] van Vliet T. , Food Emuls. Foam. Interfaces, Interact. Stab, 1999, pp. 307–317

[cit94] Dickinson E. (2012). Food Hydrocolloids.

[cit95] Zhang S., Zhang L., Bouzid M., Rocklin D. Z., Del Gado E., Mao X. (2019). Phys. Rev. Lett..

[cit96] Zaccone A., Wu H., Del Gado E. (2009). Phys. Rev. Lett..

[cit97] Whitaker K. A., Varga Z., Hsiao L. C., Solomon M. J., Swan J. W., Furst E. M. (2019). Nat. Commun..

[cit98] Flory P. J. (1985). Polym. J..

[cit99] Burla F., Dussi S., Martinez-Torres C., Tauber J., van der Gucht J., Koenderink G. H. (2020). Proc. Natl.
Acad. Sci. U. S. A..

[cit100] Park B., Yoon S., Choi Y., Jang J., Park S., Choi J. (2020). Pharmaceutics.

[cit101] Pagureva N., Tcholakova S., Rusanova K., Denkov N., Dimitrova T. (2016). Colloids Surf., A.

[cit102] Borden M. A., Longo M. L. (2002). Langmuir.

