# Supplementary material for: Structural and mechanical properties of folded protein hydrogels with embedded microbubbles
Source: Biomater Sci. 2023 Feb 8;11(8):2726–37. doi: 10.1039/d2bm01918c (PMC10088474; doi:10.1039/d2bm01918c)
Supplement: BM-011-D2BM01918C-s001 [file BM-011-D2BM01918C-s001.pdf]

Electronic Supplementary Material (ESI) for Biomaterials Science.
This journal is © The Royal Society of Chemistry 2023

Supplementary Information

Structural and mechanical properties of folded protein hydrogels with embedded microbubbles

Christa P. Brown,[a] Matt D. G. Hughes,[a] Najet Mahmoudi,[b] David J. Brockwell,[c,d] P. Louise Coletta,[d] Sally Peyman,[a] Stephen Evans[a] and Lorna Dougan[a,c]

a. School of Physics and Astronomy, Faculty of Engineering and Physical Sciences, University of Leeds, Leeds, UK.

b. ISIS Neutron and Muon Spallation Source, STFC Rutherford Appleton Laboratory, Oxfordshire, UK

c. Astbury Centre for Structural Molecular Biology, University of Leeds, Leeds, UK.

d. School of Molecular and Cellular Biology, Faculty of Biological Sciences, University of Leeds

e. Leeds Institute of Medical Research, Wellcome Trust Brenner Building, St James's University Hospital, Leeds, UK

Microbubble Population and Stability

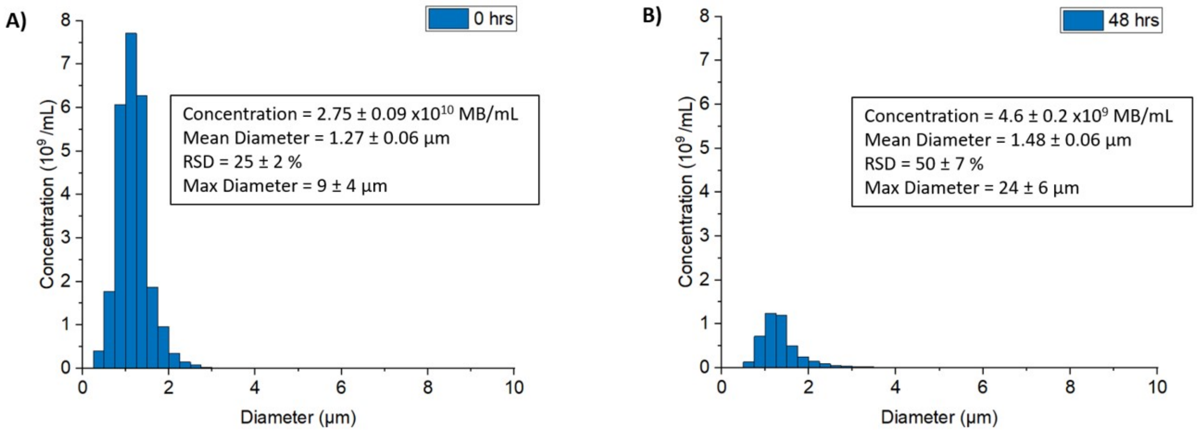

SI Figure 1: Microbubble size distribution from 45 images taken from 3 separate experimental repeat (15 images per experimental repeat) for A) at time = 0 hrs and B) at time = 48 hrs. Histogram bin width was 0.25 μm.

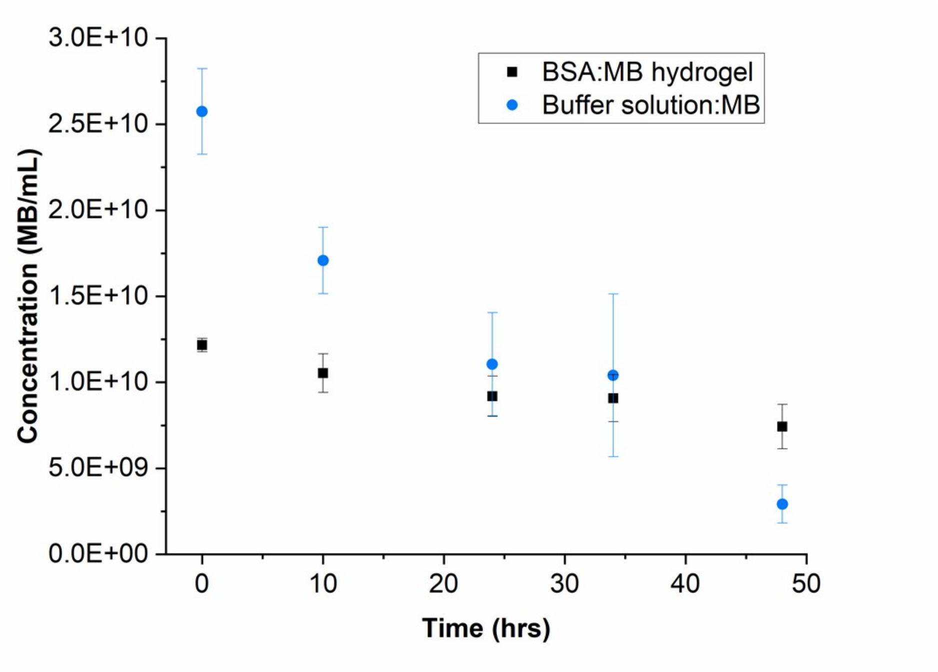

SI Figure 2: The concentration of MBs over a 48 hour period for both the MBs stored in the BSA hydrogel (black) and MBs stored in buffer solution (blue). The error bars represent standard error of the experimental repeats.

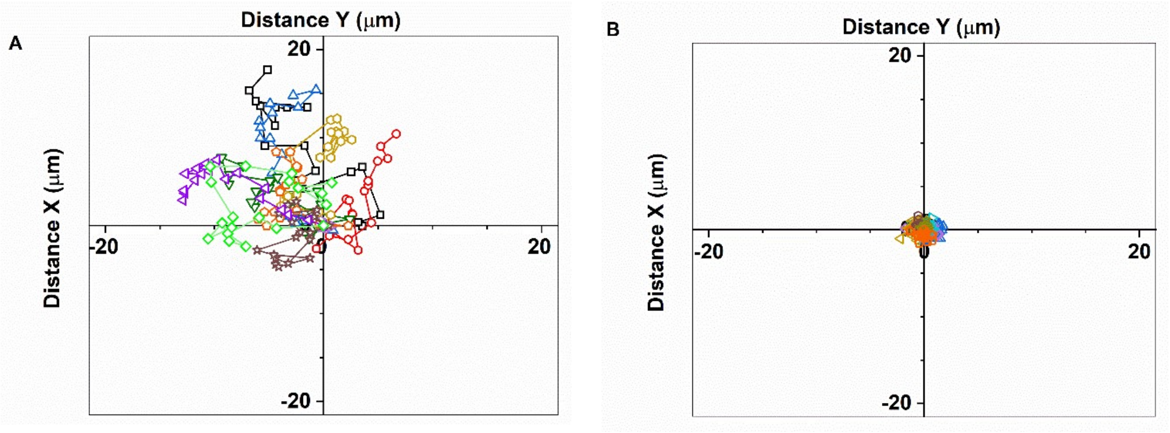

SI Figure 3: The path of 10 trajectories for A) MBs in buffer solution (sodium phosphate with 1% glycerol) compared to B) MBs in the BSA hydrogel. Optical images for both were taken with an inverted microscope (Nikon 90i, Nikon, Japan) in 30 ms time steps, for a total 570 ms (19 time steps). In A) the mean squared displacement (MSD) was determined to be 3 ± 1 x10^{-11} m^2 in X direction and 9 ± 3 x10^{-11} m^2 in the Y direction. The difference in X and Y direction arises due to a drift in the velocity. The resultant diffusion coefficients (D) were 2 ± 1 x10^{-11} m^2s^{-1} and 8 ± 3 x10^{-11} m^2s^{-1} in the X and Y directions. In B) the MSD in the X and Y direction are 6 ± 2 x10^{-13} and 7 ± 2 x10^{-13} m^2, and D in the X and Y direction are 1.1 ± 0.3 x10^{-13} m^2s^{-1} and 1.2 ± 0.4 x10^{-13} m^2s^{-1}. The movement of MBs is significantly reduced when embedded in the BSA hydrogel.

Diffusion of MBs in buffer and in the BSA hydrogel

Full gelation curve fits of rheology data

SI Figure 4: Examples of the raw gelation curves characterised with rheology (as described in the main text) for BSA (red) and BSA:MB (blue) hydrogels. Different fitting parameters using SI Equation 1 were compared where A) fits the gelation curve with one exponential component and B) fits the gelation curve with two exponential components. The results of the fits are summarised in SI Table 1.

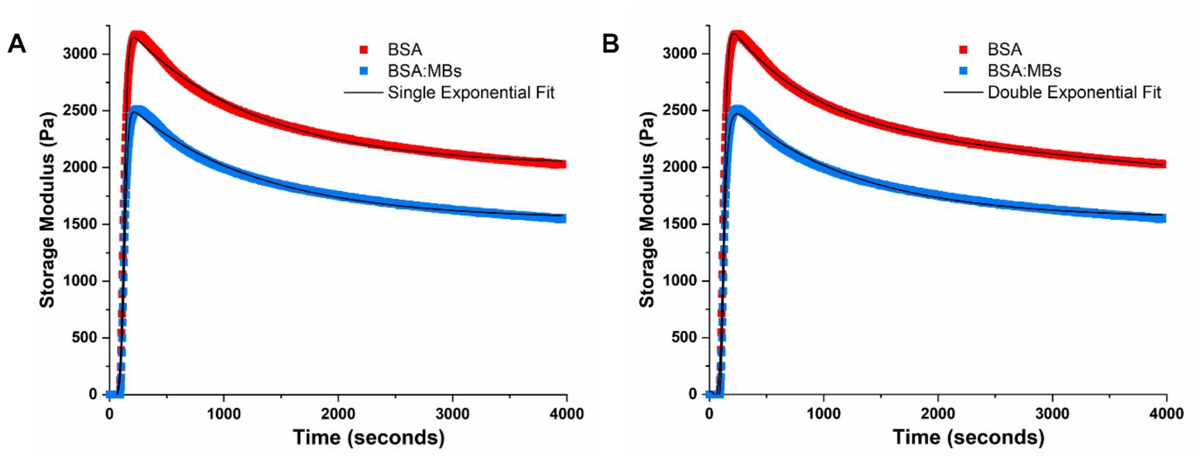

$$G'(t) = \left(1 + e^{-C(t-t_0)}\right)^{-1} \cdot \left(G'_{\infty} + B_1 e^{-t/\tau_1} + B_2 e^{-t/\tau_2}\right) + G'_0 \qquad \textbf{(1)}$$

	BSA hydrogel	BSA:MB hydrogel	BSA hydrogel	BSA:MB hydrogel
No. of exponential components	1	1	2	2
G'_{∞} (Pa)	2084 ± 7	1594 ± 6	-329543 ± 8048818	1513 ± 7.36679
B_1	1385 ± 4	1163 ± 4	1155 ± 9	-39753 ± 2342
B_2	/	/	331966 ± 8048815	1192 ± 3

C	624 ± 6 x10^{-4}	629 ± 6 x10^{-4}	60 ± 5 E-4	72.92 ± 0.001
t_0 (s)	127.2 ± 0.2	131.6 ± 0.6	127.9 ± 0.2	116.0 ± 0.5
τ_1 (s)	1112 ± 9	1113 ± 9	741 ± 14	33.9 ± 0.5
τ_2 (s)	1 ± 0	1 ± 0	3974181 ± 9.69 9E7	1061 ± 7
G_0 (Pa)	-67 ± 6	-49 ± 5	-74 ± 6	40 ± 7
Adj. R-Square	0.99625	0.99616	0.99708	0.99763

Table 1: Fitting values with SI Equation 1 compared for one exponential constant to two exponential constants for the gelation curves in SI Figure 4. The success of the fitting can be quantified by comparing the resultant values from the fit to the data. For example, the sum of constants B_1 and B_2 is theoretically equivalent to the difference between the peak in G' and G'_∞. When fitting with two exponential constants in this example, the BSA hydrogel results in a negative value for G'_∞ and the BSA:MB hydrogel results in a negative value for B_1, which suggests that use of two exponential constants is unsuitable for fitting the gelation curves for BSA and BSA:MB hydrogels

Time constant for relaxation of the gelation curves for BSA and BSA:MB hydrogels

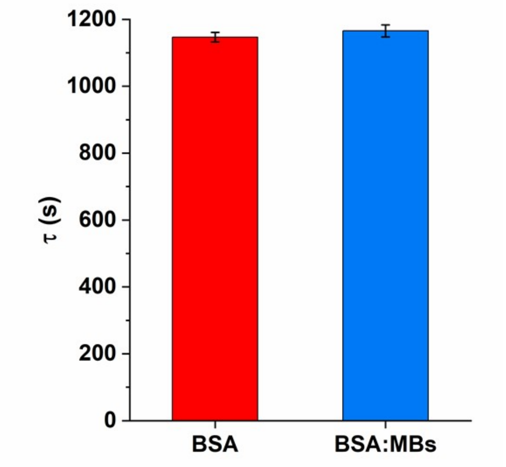

SI Figure 5: Time constant, τ, was determined from the average gelation curve fits of the data shown in Figure 5 in the main manuscript, using Equation 1 in the main manuscript, shown in the absence (red) and presence of MBs (blue).

Linear fits of the gelation curves.

	BSA hydrogel	BSA:MB hydrogel
K_{max} (Pa s^{-1})	55.8 ± 66.9	38.0 ± 0.9
C (Pa)	-5320 ± 70	-3700 ± 100
Adj. R-Square	0.9989	0.99158

Table 2: Linear fit results for the BSA and BSA:MB hydrogel from the application of a linear fit. The linear fit used was $G' = k_{max}t + C$, where C is where linear fit would be if extrapolated to t = 0 s.

Efficiency of photo-activated cross-linking reaction for BSA proteins

To quantify the number of dityrosine bonds formed in the chemically crosslinked in the hydrogel is broken down with acid and the fluorescence emission of dityrosine bonds assessed. Firstly, 4 mL of the pre-gel solution is pipetted into a 2 mL centrifuge tube with Pipetman Diamond (D10) pipette tips (Gilson, USA) to ensure accurate pipetting of low volumes. The pre-gel solution is irradiated

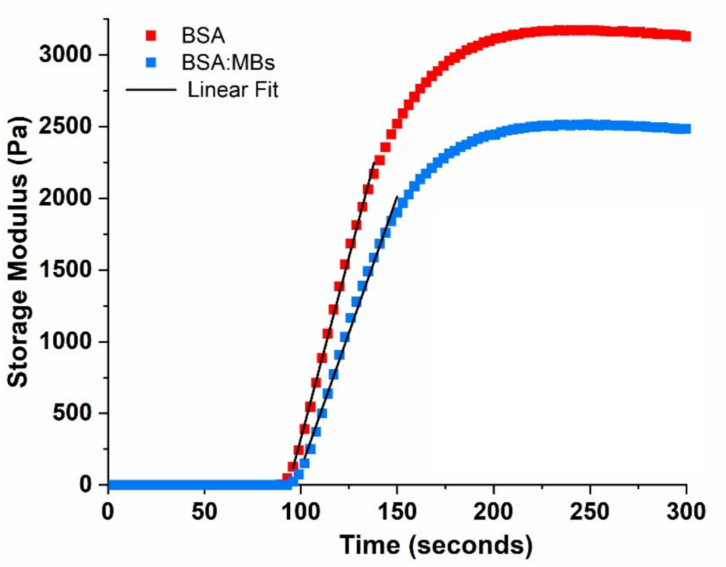

SI Figure 6: Gelation curves for BSA (red) and BSA:MB hydrogels (blue), showing only the first 300 s, where the LED lamp that initiates the cross-linking procedure is turned on at t = 60s. and turned off at t = 660 s. Example of the linear fits after the lamp is turned on, to determine the gradient of the linear region, k_{max}, for both hydrogel systems. SI Table 2 summarises the fitting

under lamp for 5 mins to gel the sample. To digest the hydrogel, 2 mL of 6 M HCl is added and boiled at 105°C for 2 hours. The acid hydrolysis product was neutralised by adding 120 mL of 5 M NaOH to 100 mL of the acid, and diluted in Na_2CO_3-$NaHCO_3$ buffer.[1]

Cuvette-based steady-state fluorescence emission spectroscopy measurements were performed on an Edinburgh Instruments FLS980 fluorescence spectrophotometer. All samples were temperature controlled to 20°C during the measurements. Quartz cuvettes were used to hold 3 mL of the sample. Samples were excited with a 450 W Xenon lamp at 315 nm, the excitation wavelength for dityrosine bonds. An emission spectrum was produced from collecting the emission from 330 - 600 nm with a red-sensitive photomultiplier tube (PMT, Hamamatsu R928 PMT) used for detection of light. A 4 nm bandwidth was used for both excitation and emission slits, which controls the amount of light passing through from the Xe lamp and to the PMT. The emission data was collected in steps of 1 nm, with a dwell time of 0.1 s, with 5 scans averaged for each sample to improve the signal to noise

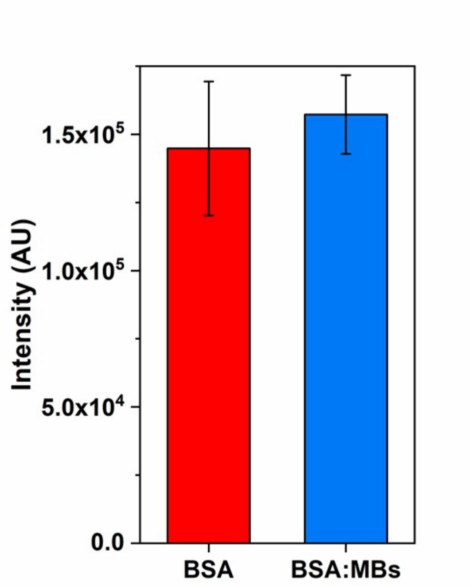

SI Figure 7: BSA hydrogel in the absence (red) and presence of MBs (blue) both excited at 315 nm and emission taken at 415 nm characteristic of the excitation and emission from dityrosine covalent bonds. The fluorescence emission intensity at 415 nm is compared. The error bars are the standard error of three experimental repeats.

ratio. The data was compared at an emission of 415 nm.

Frequency Sweep

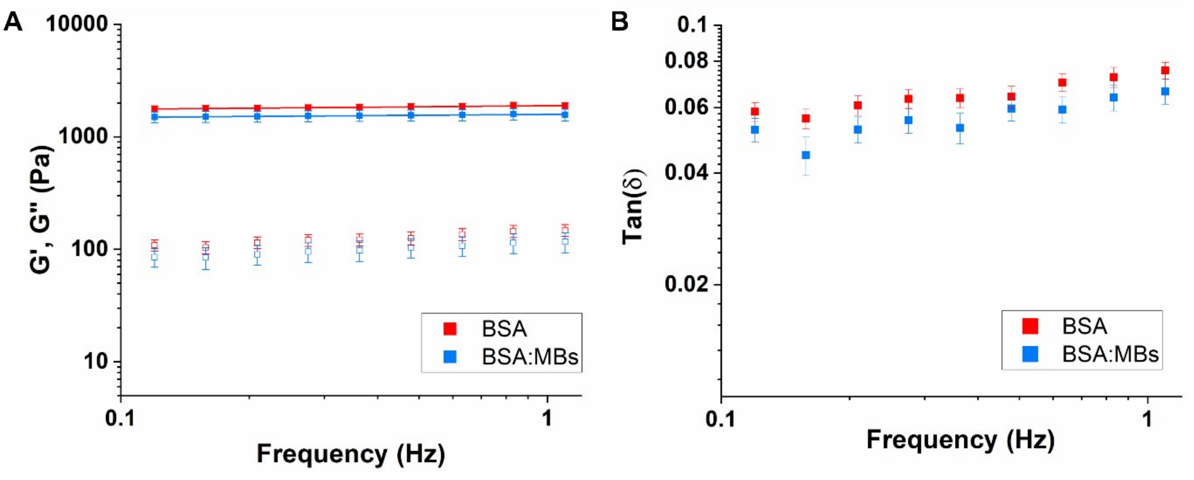

SI Figure 8: A) Frequency sweep for BSA hydrogels in the absence (red) and presence of MBs (blue) for both the storage modulus, G' (closed squares) and the loss modulus, G'' (open squares). B) The loss ratio for the BSA hydrogel in the absence (red) and presence of MBs

Stress-Strain Curves

SI Figure 9: A) Stress strain curve in the absence (red) and presence of MBs (blue). B) A storage modulus (G') was calculated from the linear region of the stress strain curve. C) The energy dissipated when loading and unloading a shear strain, and efficiency of the loading and unloading cycle in the absence and presence of MBs, where the efficiency was determined with SI Equation 2.

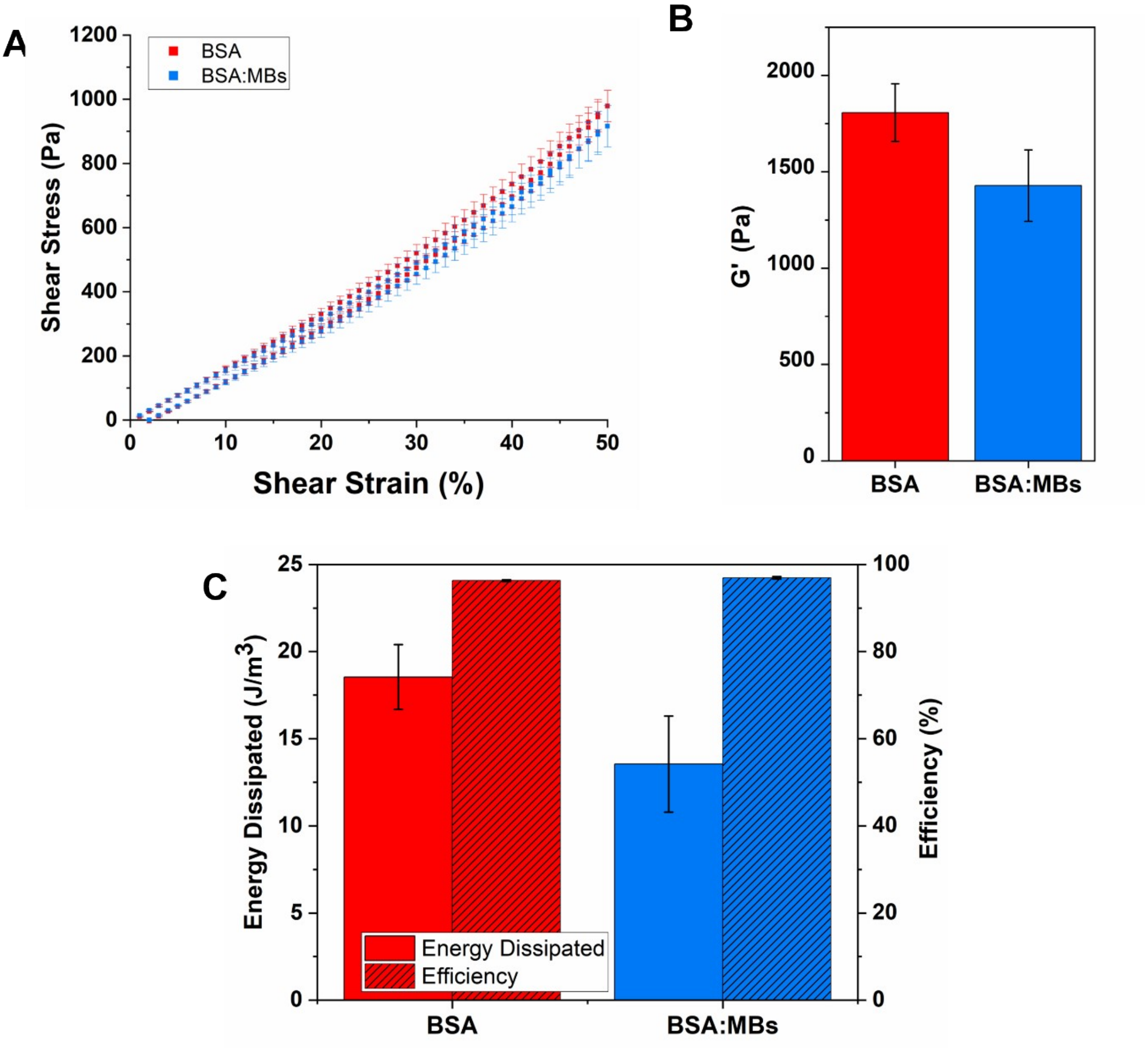

$$Eff = 1 - \frac{Energy\ Dissipated}{Strain_{Max} \cdot Stress_{Max}} \qquad \textit{Equation 2}$$

Where *Eff* is the efficiency, $strain_{max}$ is the maximum shear strain that is applied to the hydrogel, $stress_{max}$ is the shear stress recorded at the maximum shear strain.[2]

References

1 J. Fang and H. Li, *Langmuir*, 2012, **28**, 8260–8265.

2 M. D. G. Hughes, B. S. Hanson, S. Cussons, N. Mahmoudi, D. J. Brockwell and L. Dougan, *ACS Nano*, 2021, **15**, 11296–11308.
